# ZNF652-Induced circRHOT1 Promotes SMAD5 Expression to Modulate Tumorigenic Properties and Nature Killer Cell-Mediated Toxicity in Bladder Cancer via Targeting miR-3666

**DOI:** 10.1155/2021/7608178

**Published:** 2021-12-09

**Authors:** Hu Ke, Jiabin Zhang, Fei Wang, Yunhe Xiong

**Affiliations:** ^1^Urology Department, Renmin Hospital of Wuhan University, Wuhan, 430060 Hubei Province, China; ^2^Urology Department, Mindong Hospital Affiliated to Fujian Medical University, No. 89 Heshan Street, Fu'an, Fujian, China; ^3^Urology Department, The Third Clinical Medical College of China Three Gorges University, Gezhouba Central Hospital of Sinopharm, Yichang, Hubei, China

## Abstract

Bladder cancer (BC) is the 9^th^ most frequent diagnosed tumor and the 2^nd^ most common urology tumor worldwide. Despite the considerable advancement that BC treatment has made recently, the five-year survival rate of BC remains unsatisfactory. Novel therapeutic strategies for BC clinical intervention are therefore urgently needed now more than ever. circRHOT1 is a newly identified circRNA that plays a crucial role in multiple types of tumorigeneses. However, it remains unclear whether circRHOT1 plays a functional role in BC progression. Our findings suggest that circRHOT1 was highly expressed in BC tumor tissues and cell lines. The results from CCK-8, EDU, Transwell migration, and NK cell-mediated cytotoxicity detection assays suggested that circRHOT1 knockdown could markedly suppress BC cell proliferation and migration level and could aggravate the sensitivity of BC cells to NK cells. Subsequently, we conducted bioinformatics analysis followed by RNA pull-down, ChIP, and luciferase reporter assays, from which we found that circRHOT1 expression in BC cells could be regulated by ZNF652, and circRHOT1 could promote SMAD5 expression to regulate BC cell cellular progression by sponging miR-3666. These results may provide a new direction for developing novel diagnostic or therapeutic targets for BC.

## 1. Introduction

Bladder cancer (BC) has been demonstrated to be the 9^th^ most frequently diagnosed cancer worldwide and the 2nd most common tumor in the urology system. Nearly 30% of BC patients have muscle-invasive BC, thus are vulnerable to distance metastasis [1]. In 2018, more than 540,000 patients were newly diagnosed as having BC, of which approximately 200,000 lost their lives due to BC [2]. The mortality rate of BC worldwide has been thought to be a considerable problem of the world health care system [3]. Despite the recent development of remarkably advanced BC treatment, the five-year survival rate of BC remains unsatisfactory [4, 5]. Thus, a novel therapeutic strategy for BC clinical intervention is now more urgently needed than ever.

Circular RNAs (circRNAs), which belong to one of the noncoding RNA subtypes, are spliced from the process of RNA transcriptional production and formed by a covalently closed loop [6]. With the innovation of high-throughput technology in the past decades, the characterizations of circRNAs have been well documented. circRNAs are abundantly expressed and highly conserved in a tissue-specific manner [7]. A number of studies have revealed that circRNAs play a functional role in various types of tumorigenesis by acting as a molecular sponge for miRNAs or interacting with RNA-binding proteins (RBPs) [8–10].

With the structural and mechanistic findings of circRNAs in recently years, the functional roles of circRNAs in multiple biological or pathological progressions have been demonstrated, especially in the initiation or progression of numerous types of tumorigenesis [11–14]. In BC, circRIP2 can promote the progression of BC through the miR-1305/Tgf-*β*2/smad3 pathway [15]. circACVR2A inhibits the tumorigenic properties of BC by acting as a sponge for miR-626 [16]. Hsa_circ_0001361 aggravates BC progression via the miR-491-5p/MMP9 axis [17]. Increasing evidence has indicated the important role of circRNAs in BC progression.

Circular RNA RHOT1 (hsa_circRNA_102034), which is spliced from the RHOT1 gene, plays an oncogenic role in hepatocellular carcinoma progression by initiating the expression of NR2F6 to recruit TIP60 [18]. circRHOT1 also has a functional role in breast cancer and pancreatic cancer by acting as a molecular miRNA sponge [19–21]. As one of the newly identified circRNAs, circRHOT1 has a vital role in multiple types of tumorigeneses, but its involvement in the progression of BC remains unknown.

In this study, we hypothesized that circRHTO1 may have a functional role in BC progression. Our findings suggested that the downregulation of circRHOT1 inhibited the proliferation and migration level of BC cells, while promoting NK cell-mediated cytotoxicity to BC cells. Furthermore, the upstream and downstream mechanisms of circRHOT1 in BC were investigated. We found a novel ZNF652/circRHOT1/miR-3666/SMAD5 axis in BC development. This study may provide new and promising directions for the development of new diagnostic or therapeutic targets for BC.

## 2. Materials and Methods

### 2.1. Cell Culture and Transfection

Bladder cancer cell lines (253J, BT-B, HTB9, HT-1376, 5637, T24, and UMUC3) and the normal human bladder cell SV-HUC-1 were commercially obtained from the Shanghai Institute of Cell Biology (Shanghai, China). The cells were cultured using RPMI-1640 medium supplemented with 10% FBS (Sigma-Aldrich, USA) at 37°C in a humid atmosphere containing 5% CO_2_. The cells were passaged at every 48-72 h. All the designed siRNAs, lentiviruses, mimics, and inhibitors were purchased from GeneChem (Shanghai, China). The siRNA sequences of circRHOT1 was as follows: si-circRHOT1#1: 5′-CAG CAG GUU CCU CCC CGG GTT-3′, si-circRHOT1#2: 5′-ACA GCA GGU UCC UCC CCG GTT-3′, and si-NC: 5′-UUC UUC GAA CGU GUC ACG UTT-3′. The transfections were conducted using the Lipofectamine 3000 reagent (Thermo Fisher) following the manufacturer's guide.

### 2.2. Quantitative Real-Time Polymerase Chain Reaction (qRT-PCR)

RNAs from BC tissues and cells were extracted by the TRIzol method (15596026, Invitrogen, USA) treatment. After being qualitied and concentrated, RNAs were transcribed into cDNA using a reverse transcription kit (RR047A, Takara, Japan). Subsequently, the relative RNA expressions were measured by qRT-PCR using an SYBR Premix EX Taq kit (RR420A, Takara). The glyceraldehyde-3-phosphate dehydrogenase (GAPDH) and U6 were used as internal controls, and the quantification was carried out using the 2−*ΔΔ*Ct method. The primers used in this study were as follows: circRHOT1: F, 5′-ATC ACC ATT CCA GCT GAT GT-3′, R, 5′-TGC TGT CTT TGT CTG TTC TTTC-3′; RHOT1: F, 5′-GGG AGG AAC CTC TTC TGGA-3′, R, 5′-ATG AAG AAA GAC GTG CGG AT-3′; ZNF652: F, 5′-GAT GCG AGA ACT GTG ACG AA-3′, R, 5′-GAA ATC CTT GCC ACA CCA CT-3′; miR-3666: F, 5′-ACG AGA CGA CGA CAG AC-3′, R, 5′-CAG TGC AAG TGT AGA TGC CGA-3′; SMAD5: F, 5′-CGG TAG CCA ACT GAC TTT GAGT-3′, R, 5′-ACC TTG TTT CCA GCC CAA CA-3′; GAPDH: F, 5′-ACC ACA GTC CAT GCC ATC AC-3′, R, 5′-TCC ACC ACC CTG TTG CTG TA-3′; U6; F, 5′-GCU UCG GCA GCA CAU AUA CUA AAAU-3′, R, 5′-CGC UUC ACG AAU UUG CGU GUC AU-3′.

### 2.3. Western Blot

The protein expression of SMAD5 protein in transfected HT-1376 and HTB9 cells was detected by using western blot. Briefly, SMAD5 protein was extracted from the transfected BC cells using a lysis buffer containing protease and phosphatase inhibitors. The protein was separated by SDS-PAGE and then electronically transferred to a polyvinylidene fluoride membrane. The membrane was incubated with primary antibody against SMAD5 (CST, 12534S, 1 : 1000), followed by the corresponding secondary antibody (CST, 1 : 3000, 7074S). GAPDH (CST, 5174S, 1 : 1000) was applied as the control group. The resultant membrane was photographed using a chemiluminescence reagent (Advansta, USA) and analyzed with ImageJ software (BIO-RAD, USA).

### 2.4. Cell Proliferation Assay

For the Cell Counting Kit 8 (CCK-8) assay, transfected HT-1376 and HTB9 cells after transfection were seeded into a 96-well plate (1 × 10^4^ cells per well) and were cultured in DMEM. After two days, 10 *μ*L of CCK-8 solution (Dojindo, Japan) was added to the cells and incubated for 120 min. An absorbance at 450 nm of the cells was measured. EDU assay was carried out using a Cell-Light EdU DNA Cell Proliferation Kit procured from RiboBio (Guangzhou, China) according to the manufacturer's guideline. The results were photographed using an Olympus FSX100 microscope (Tokyo, Japan). The proliferation level was assessed by calculating the radio of EDU-stained cells.

### 2.5. Cell Migration Assay

The Transwell assay was conducted to assess the migration level of transfected HT-1376 and HTB9 cells.

The lower chamber was filled with a culture medium containing 10% FBS (Gibco), whereas the upper chamber was seeded with 1 × 10^5^ transfected cells and then filled with nonserum medium. After two days, 4% paraformaldehyde was added to the upper chamber and incubated for 10 min; after that, it was fixed with 0.1% crystal violet for 10 min. Finally, the migrated cells were observed and recorded.

### 2.6. Natural Killer Cell Cytotoxicity Assay

Natural killer cell cytotoxicity to the transfected HT-1376 and HTB9 cells was detected using calcein release assay and perforin polarization assay and conjugation assay as a published research description [22]. (A brief introduction of experimental process: Firstly, HT-1376 and HTB9 cells were incubated with NK cells (1 : 1) at 37°C for 30 min. Subsequently, the mixed cells were incubated in 12-well plates supplemented with poly-D-lysine-coated slides at 37°C for 60 min. Next, the slides were retrieved and then added with 4% paraformaldehyde, followed by a permeabilizing agent (0.5% Triton X-100 in PBS). After that, they were incubated with antiperforin antibody and Alexa Fluor 568-conjugated secondary antibody. Finally, the results were visualized under a confocal microscope.)

### 2.7. RNA Pull-Down Assay

Biotinylated RNA probes were designed and commercially obtained from GeneChem (Shanghai, China). The experiment was conducted following the guideline supplied by the manufacturer. In brief, HT-1376 and HTB9 cells at 50% confluence were incubated with biotinylated probes (50 nM) for 24 h and were lysed thereafter. Next, streptavidin magnetic beads (Life Technologies) were added into the cell lysates and incubated for 3 h. A TRIzol reagent was used to isolate RNA from interacting bounds, and the results were analyzed by qRT-PCR assay. The experiment was conducted in triplicate.

### 2.8. Chromatin Immunoprecipitation (ChIP) Assay

ChIP assay was conducted using a chromatin immunoprecipitation kit (Thermo Scientific) according to the manufacturer's guideline. Firstly, chromatin from the lysate of transfected cells was isolated and then digested with micrococcal nuclease to generate DNA fragments. Next, the antibodies were subjected to immunoprecipitation assay. The results were analyzed by qRT-PCR. Each experiment was repeated three times.

### 2.9. Luciferase Reporter Assay

The position of putative binding sites were cloned into the luciferase reporter vectors (Synbio Technologies, Suzhou, China), which were then cotransfected with indicated mimics or siRNAs into HT-1376 and HTB9 cells. Relative luciferase activities were measured and analyzed following the manufacturer's instruction (E292, Promega, USA).

### 2.10. Statistical Analysis

GraphPad version 5.0 was used to statistically analyze the data. The differences between two independent groups were analyzed using the Student *t*-test. The differences between multiple groups were determined using one-way ANOVA test. *P* < 0.05 was considered significantly different. All experiments were performed in triplicate.

## 3. Results

### 3.1. Characterization of circRHOT1 in BC

**Figure 1:**
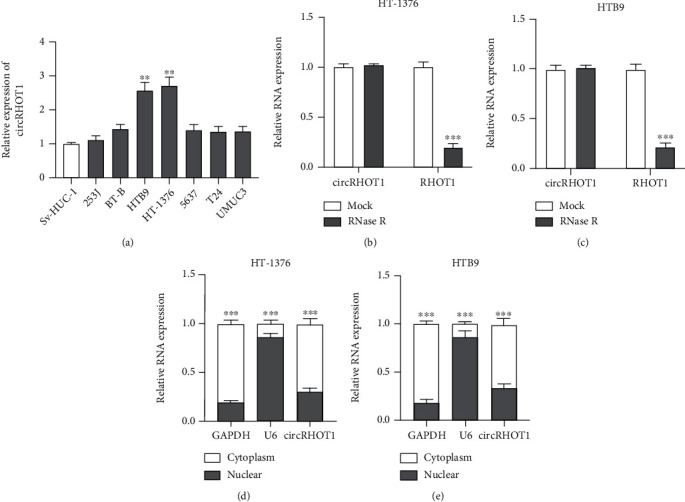
Characterization of circRHOT1 in BC. (a) circRHOT1 level in BC cell lines detected by qRT-PCR. (b, c) circRHOT1 and RHOT1 mRNA expression measured by qRT-PCR in HT-1376 (b) and HTB9 (c) cells treated with RNase R or mock. (d, e) circRHOT1 distribution detected by cellular fragment assay in HT-1376 (d) and HTB9 (e) cells. Experiments were conducted in triplicate. ^∗∗^*P* < 0.01 and ^∗∗∗^*P* < 0.001.

To determine whether circRHOT1 is involved in the development of BC, we first examined the expression of circRHOT1 in BC cell lines. As shown in Figure 1(a), the expression of circRHOT1 was significantly increased in BC cells, especially in HTB9 and HT-1376 cells. Subsequently, we evaluated the circular RNA characterization of circRHOT1 in BC cells. Compared with RHOT1 mRNA, circRHOT1 could better resist the digestion by RNase R in HT-1376 (Figure 1(b)) and HTB9 (Figure 1(c)). Moreover, circRHOT1 was mainly distributed in the cytoplasm of HT-1376 (Figure 1(d)) and HTB9 (Figure 1(e)) cells. These results indicated that circRHOT1 may be involved in BC development.

### 3.2. circRHOT1 Knockdown Attenuates BC Cell Proliferation, Migration, and Susceptibility to NK Cells

**Figure 2:**
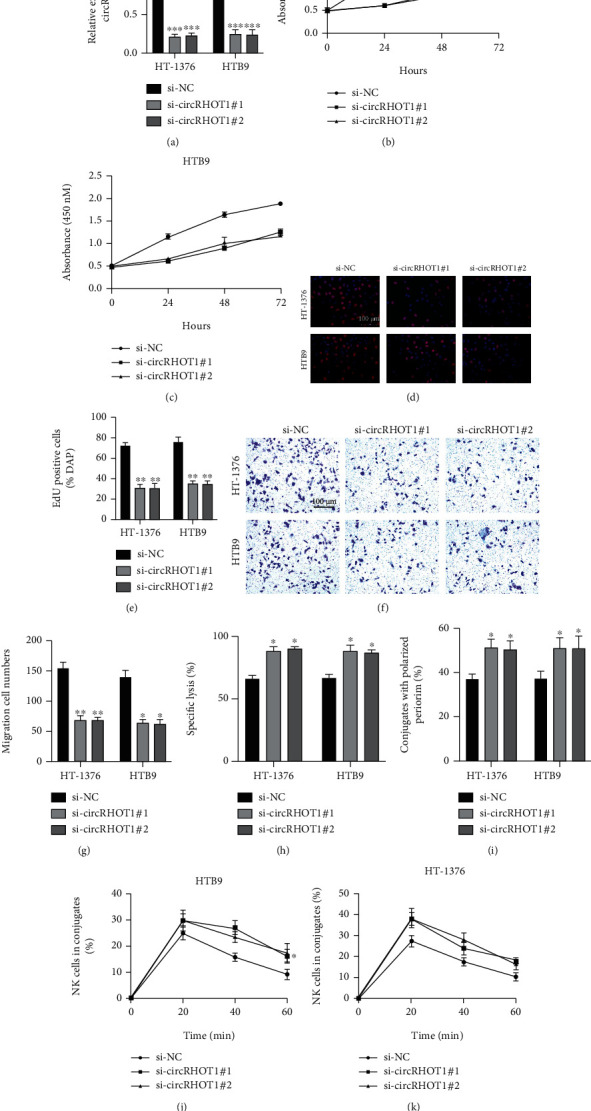
circRHOT1 knockdown attenuates BC cell proliferation, migration, and susceptibility to NK cells. (a) HT-1376 and HTB9 cells were stably transfected with si-NC, si-circRHOT1#1, and si-circRHOT1#2. Expression level of circRHOT1 in transfected cells was detected by qRT-PCR. (b, c) Proliferation of transfected HT-1376 (b) and HTB9 (c) cells determined by CCK-8 assay. (d, e) Measurement of proliferation of HT-1376 and HTB3 cells (d) with downregulated circRHOT1 by EDU assay, and analysis results (e). (f, g) Measurement of migration of HT-1376 or HTB3 cells (f) with downregulated circRHOT1 by Transwell migration assay, and statistical analysis results (g). (h) Killing of HT-1376 or HTB3 cells by circRHOT1 knockdown measured by 51Cr release assay. (i) Numbers of NK cells and circRHOT1-knockdown cells measured by perforin polarization assay. (j, k) Conjugation between NK cells and HT-1376 (j) or HTB9 cells (k) with downregulated circRHOT1 assessed by conjugation assay. Experiments were conducted in triplicate. ^∗^*P* < 0.05, ^∗∗^*P* < 0.01, and ^∗∗∗^*P* < 0.001.

To evaluate the functional role of circRHOT1 in BC, circRHOT1 siRNAs (si-NC, si-circRHOT1#1, and si-circRHOT1#2) were introduced into HT-1376 and HTB9 cells, and the results showed that circRHOT1 expression in transfected cells was efficiently downregulated (Figure 2(a)). The results from the CCK-8 assay further showed that circRHOT1 knockdown markedly suppressed the proliferation level of HT-1376 (Figure 2(b)) and HTB9 (Figure 2(c)) cells, and these observations were further confirmed by EDU assay (Figures 2(e) and 2(f)). circRHOT1 knockdown also significantly suppressed the migration level of HT-1376 and HTB9 cells (Figures 2(f) and 2(g)). Natural killer (NK) cell-mediated cytotoxicity is essential for the immune response of BC [23–25]; thus, we further investigated whether circRHOT1 can influence the susceptibility of BC cells to NK cells. The results from calcein release assay (Figure 2(h)), perforin polarization assay (Figure 2(i)), and conjugation assay (Figures 2(j) and 2(k)) showed that circRHOT1 knockdown markedly aggravated NK cell-mediated cytotoxicity towards BC cells. These findings indicated that circRHOT1 plays an oncogenic role in BC progression.

### 3.3. ZNF652 Transcriptionally Induced circRHOT1 Expression in BC Cells

**Figure 3:**
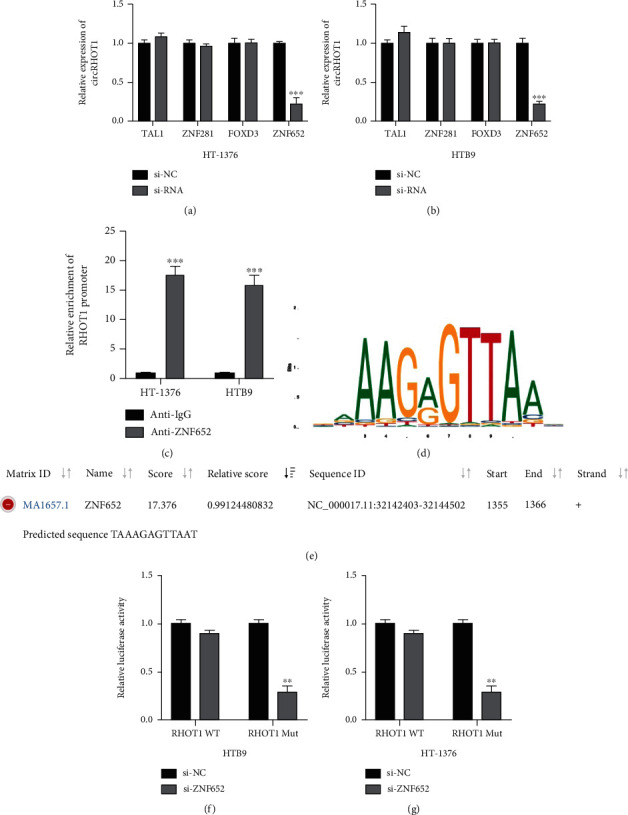
ZNF652 transcriptionally induces circRHOT1 expression in BC cells. To find the upstream regulators of circRHOT1, we conducted a search on JASPAR (http://jaspar.genereg.net/) database (Score > 600). (a, b) Relative expression of circRHOT1 detected by qRT-PCR assay in HT-1376 (a) and HTB9 (b) cells pretransfected with indicated siRNAs. (c) ChIP assay conducted to detect the enrichment of circRHOT1 promoter, followed by qRT-PCR. (d, e) The predicted binding sequences between ZNF652 and RHOT1 promoter obtained from the JASPAR dataset. (f, g) Variations of luciferase activities of RHOT1 promoter in ZNF652 knockdown HTB9 (f) and HT-1376 (g) cells. Experiments were conducted in triplicate. ^∗∗∗^*P* < 0.001.

The upstream regulator of circRHOT1 was investigated to verify the mechanism underlying the involvement of circRHOT1 in BC development. By utilizing the JASPAR dataset, four transcription factors (TAL1, ZNF281, FOXD3, and ZNF652) were identified. siRNAs of these factors were synthesized and then transfected into HT-1376 and HTB9 cells. ZNF652 knockdown was found to markedly repress circRHOT1 expression in HT-1376 (Figure 3(a)) and HTB9 (Figure 3(b)) cells. The results from the ChIP assay indicated that the RHOT1 promoter was significantly enriched in the complex with the ZNF652 antibody compared with the IgG antibody (Figure 3(c)). These findings suggested that ZNF652 may bind to the RHOT1 promoter. The binding sites between ZNF652 and the RHOT1 promoter are shown in Figures 3(d) and 3(e). Next, the luciferase activities driven by RHOT1 Mut in HTB9 (Figure 3(f)) and HT-1376 (Figure 3(g)) cells were found to be markedly reduced by ZNF652 knockdown. These findings show that ZNF652 could transcriptionally induce circRHOT1 expression in BC cells.

### 3.4. miR-3666 Is a Downstream Target of circRHOT1

**Figure 4:**
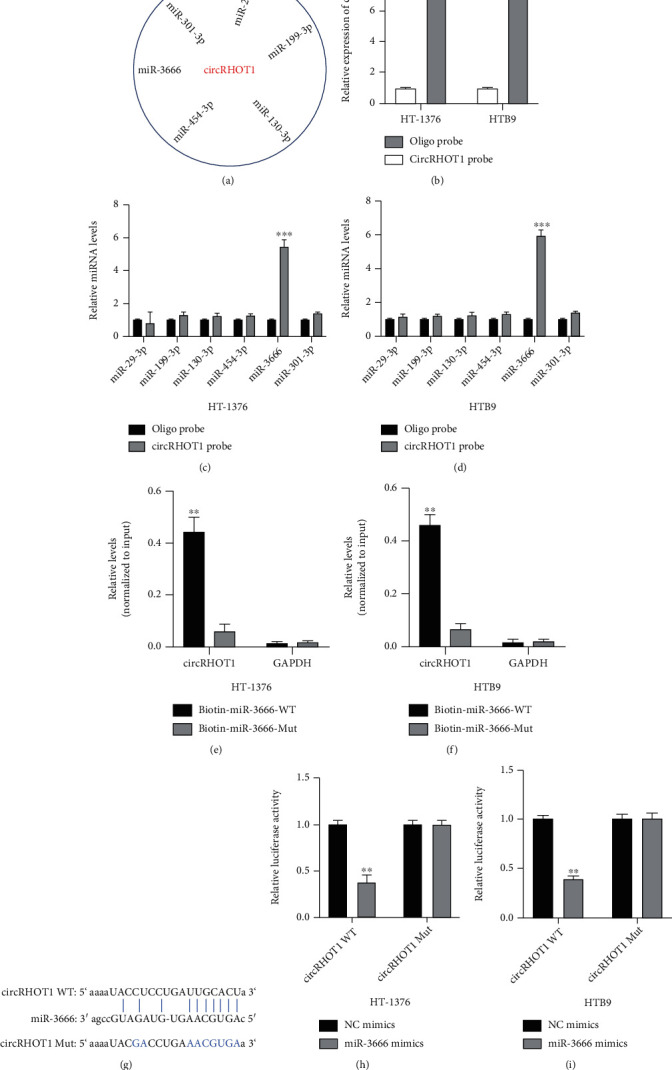
miR-3666 is a downstream target of circRHOT1. (a) The identified six putative miRNAs. (b) RNA pull-down assay conducted using circRHOT1 probe, followed by qRT-PCR, to measure the expression level of circRHOT1. (c, d) Putative miRNAs expression in HT-1376 (c) and HTB9 (d) cells measured by RNA pull-down assay using circRHOT1 probe and qRT-PCR. (e) Expression of circRHOT1 in HT-1376 (e) or HTB9 (f) cells determined by RNA pull-down assay using biotin-miR-3666-WT/Mut probe and qRT-PCR. (g) The binding sites between circRHOT1 and miR-3666 that were synthesized. (h, i) Relative luciferase activities of HT-1376 (h) and HTB9 (i) cells cotransfected with miR-3666 mimics/NC mimics and WT/Mut circRHOT1 plasmids. Experiments were conducted in triplicate. ^∗∗^*P* < 0.01 and ^∗∗∗^*P* < 0.001.

Next, the downstream mechanisms of circRHOT1 in BC progression were further investigated. The results from bioinformatics analysis results indicated that there were six putative miRNAs of circRHOT1, as depicted in Figure 4(a). The biotinylated circRHOT1 probe and oligo probe were designed and then transfected into HT-1376 and HTB9 cells, and the cell transfection efficiencies were determined (Figure 4(b)). The results from the biotinylated RNA pull-down assay showed that miR-3666 was markedly enriched in the biotinylated circRHOT1 probe in both HT-1376 (Figure 4(c)) and HTB9 (Figure 4(d)) cells. Moreover, biotinylated miR-3666-WT and miR-3666-Mut probes were designed and transfected into HT-1376 and HTB9 cells. The results showed that circRHOT1 was significantly enriched in the biotin-miR-3666-WT, but not in the biotin-miR-3666-Mut probe. The position of putative binding sites between circRHOT1-WT or Mut and miR-3666 is shown in Figure 4(g). The luciferase reporter gene assay also showed that miR-3666 mimics significantly reduced luciferase activities driven by circRHOT1-WT in both HT-1376 (Figure 4(h)) and HTB9 (Figure 4(i)) cells. These results suggested that circRHOT1 can sponge miR-3666.

### 3.5. miR-3666 Overexpression Inhibits BC Cell Proliferation, Migration, and Susceptibility to NK Cells

**Figure 5:**
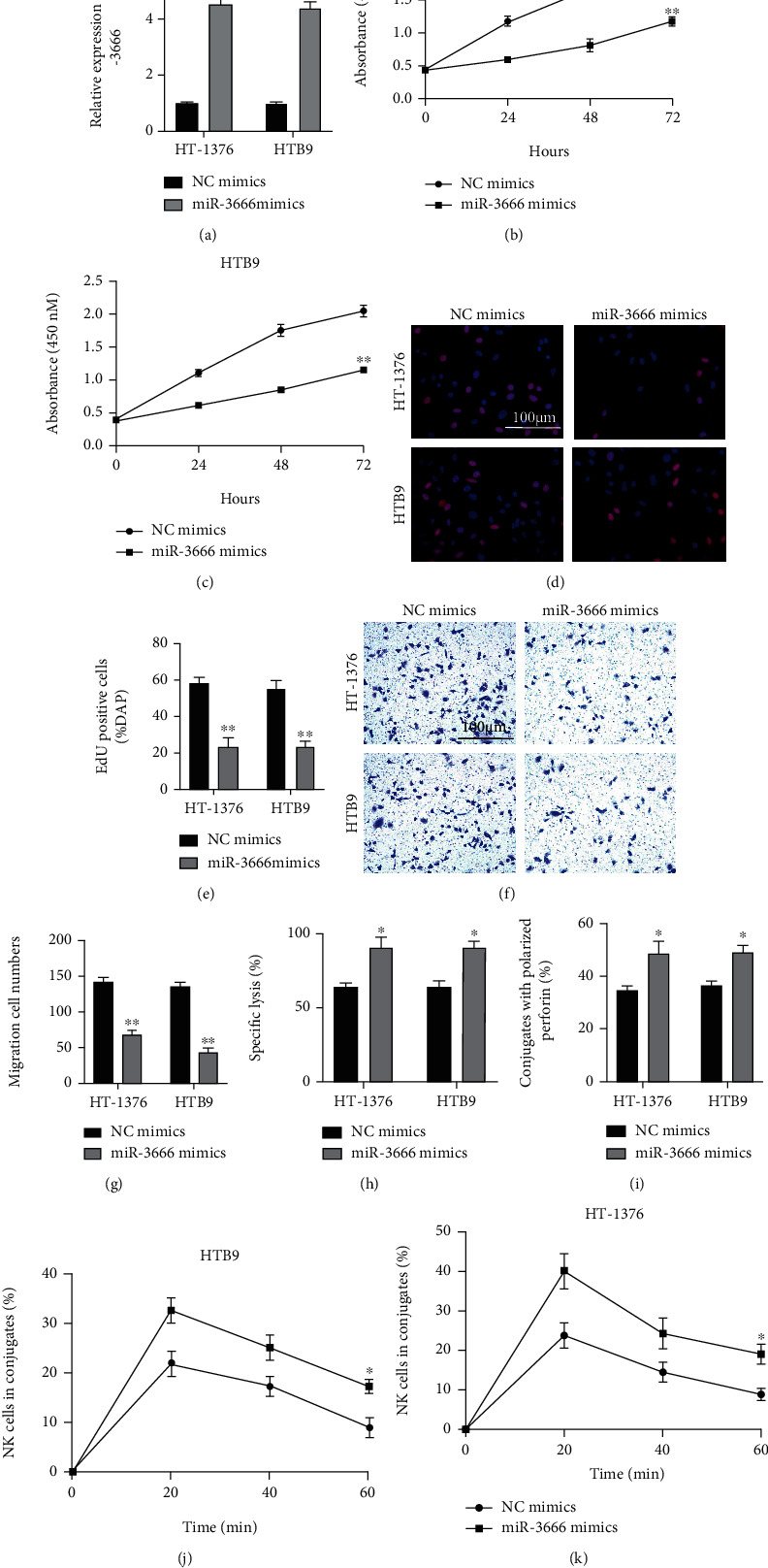
miR-3666 overexpression inhibits BC cell proliferation, migration, and susceptibility to NK cells. (a) miR-3666 level measured by qRT-PCR in BC cells transfected with miR-3666/NC mimics. (b, c) Proliferation of miR-3666-overexpressed HT-1376 (b) and HTB9 (c) cells measured by CCK-8 assay. (d, e) EDU assay performed to evaluate the proliferation of miR-3666-overexpressed HT-1376 (d) and HTB9 (e) cells. (f, g) Migration of miR-3666-overexpressed HT-1376 (f) and HTB9 (g) cells determined by Transwell migration assay. (h) The killing of HT-1376 and HTB3 cells by miR-3666 overexpression detected by 51Cr release assay. (i) Numbers of NK cells and miR-3666-overexpressed cells assessed by perforin polarization assay. (j, k) Conjugates formed between NK cells and miR-3666-overexpressed HTB9 (j) or HT-1376 cells (k) assessed by conjugation assay. Experiments were conducted in triplicate. ^∗^*P* < 0.05, ^∗∗^*P* < 0.01, and ^∗∗∗^*P* < 0.001.

The same strategies were employed to determine the functional role of miR-3666 in BC. miR-3666 mimics and normal control (NC) mimics were efficiently introduced into HT-1376 and HTB9 cells (Figure 5(a)). The overexpression of miR-3666 could markedly inhibit cell proliferation (Figures 5(b)–5(e)) and migration (Figures 5(f) and 5(g)), according to the results from the CCK-8 assay (Figures 5(b) and 5(c)), EDU assay (Figures 5(d) and 5(e)), and Transwell migration assays (Figures 5(f) and 5(g)). Further, the miR-3666 overexpression could significantly enhance the sensitivity of BC cells to NK cells, based on the observations from calcein release assay (Figure 5(h)), perforin polarization assay (Figure 5(i)), and conjugation assay (Figures 5(j) and 5(k)). These findings show that miR-3666 can inhibit the progression of BC tumorigenesis.

### 3.6. miR-3666 Can Directly Target SMAD5

**Figure 6:**
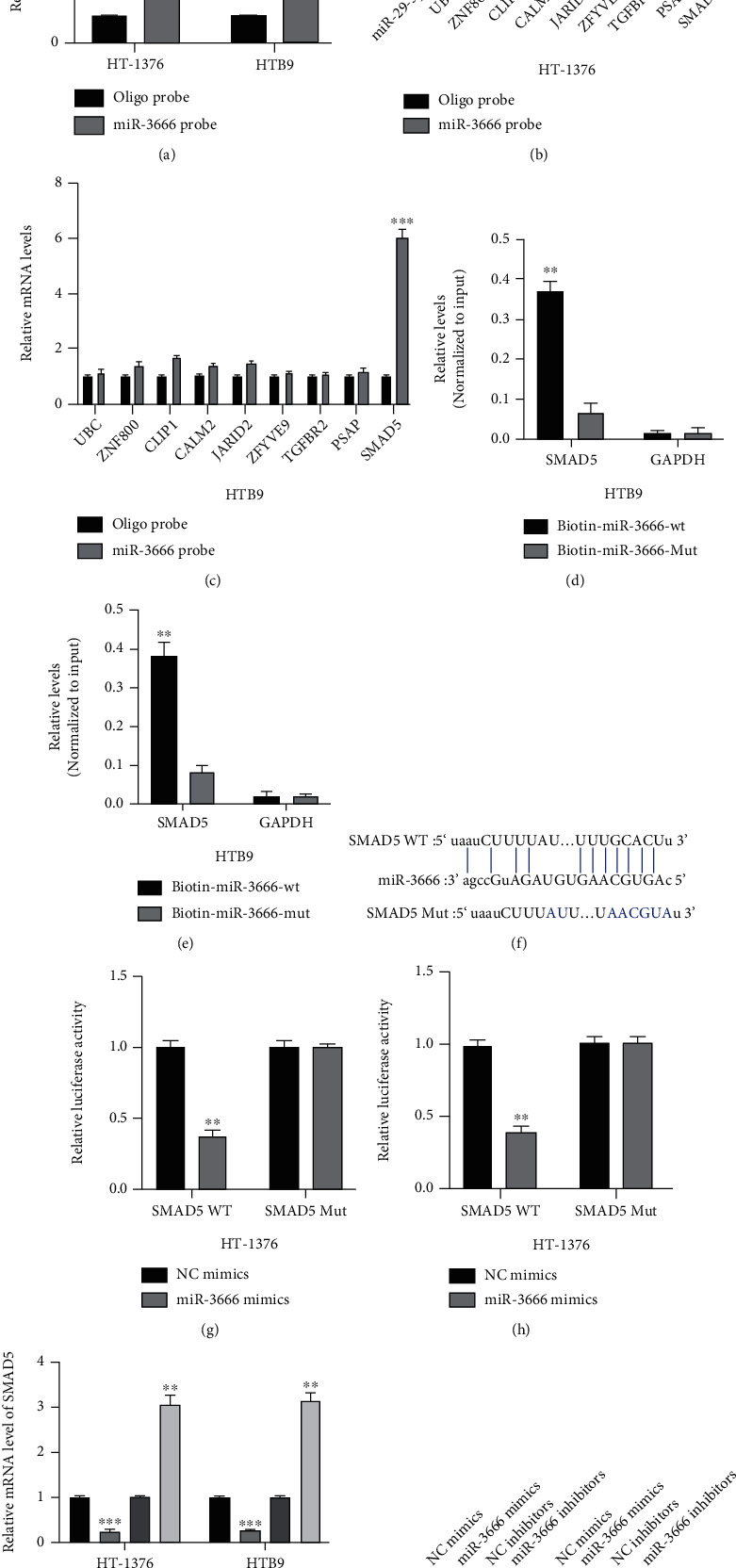
miR-3666 directly targets SMAD5. (a) RNA pull-down assay conducted using miR-3666 probe, followed by qRT-PCR, to measure miR-3666 expression. (b, c) Putative mRNA expression in HT-1376 (b) and HTB9 (c) cells determined by RNA pull-down assay using miR-3666 probe and qRT-PCR. (d, e) Expression of SMAD5 in HTB9 (d) and HT-1376 (e) cells measured by RNA pull-down assay using biotin-miR-3666-WT/Mut probe combined with qRT-PCR. (f) The binding sites between SMAD5 and miR-3666. (g, h) Relative luciferase activities in HT-1376 (g) and HTB9 (h) cells cotransfected with miR-3666 mimics/NC mimics and WT/Mut SMAD5 plasmids. (i) Relative mRNA expression of SMAD5 in HT-1376 or HTB9 cells transfected with miR-3666 mimics or inhibitors measured by qRT-PCR. (j) Expression of SMAD5 protein in HT-1376 or HTB9 cells transfected with miR-3666 mimics or inhibitors detected by western blot. Experiments were conducted in triplicate. ^∗∗^*P* < 0.01 and ^∗∗∗^*P* < 0.001.

The underlying mechanism of miR-3666 in BC was investigated. Putative downstream targets of miR-3666 were predicted by microT (http://www.microrna.gr/microT), miRmap (http://mirnamap.mbc.nctu.edu.tw/), PicTar (http://www.pictar.org/), and TargetScan (http://www.targetscan.org/) with CLIP Data: strict stringency (≥5) and Ago Exp Num ≥ 40. The biotinylated miR-3666 probe and oligo probe were designed and efficiently transfected into HT-1376 and HTB9 cells (Figure 6(a)). According to the results, SMAD5 was significantly enriched in the biotinylated miR-3666 probe in both HT-1376 (Figure 6(b)) and HTB9 (Figure 6(c)) cells. Moreover, SMAD5 was also significantly enriched in the biotin-miR-3666-WT, but not in the bio-miR-3666-Mut probe in both HTB9 (Figure 6(d)) and HT-1376 (Figure 6(e)) cells. The position of putative binding sites between SMAD5 and miR-3666 is shown in Figure 6(f). miR-3666 overexpression markedly repressed the luciferase activities driven by SMAD5 WT in either HT-1376 cells (Figure 6(g)) or HTB9 cells (Figure 6(h)). The results from qRT-PCR and western blot assays showed that miR-3666 could negatively regulate the expression of SMAD5 in BC cells (Figures 6(i) and 6(j)). These findings demonstrated that miR-3666 can sponge SMAD5 and negatively regulates SMAD5 expression in BC cells.

### 3.7. SMAD5 Is Essential for the Functional Role of circRHOT1 in BC

**Figure 7:**
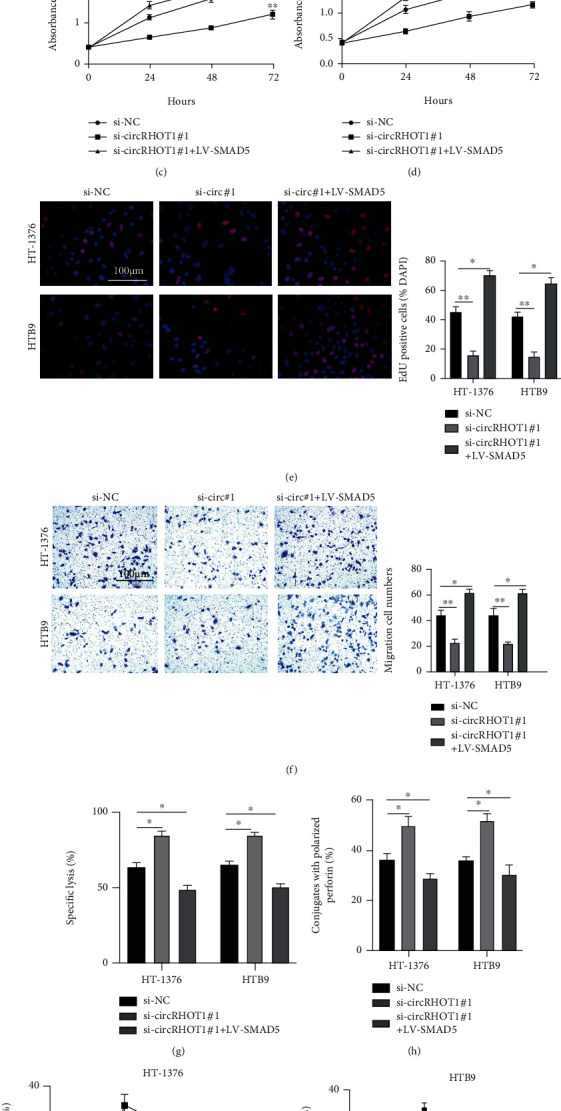
SMAD5 is essential for the functional role of circRHOT1 in BC. (a) Expression level of circRHOT1 in transfected cells measured by qRT-PCR. (b) Expression of SMAD5 in transfected cells detected by qRT-PCR. (c, d) Proliferation of transfected HT-1376 (c) and HTB9 (d) cells detected by CCK-8 assay. (e) Proliferation of transfected HT-1376 and HTB9 cells determined by EDU assay. (f) Migration of transfected HT-1376 and HTB9 cells detected by Transwell migration assay. (g) The killing of HT-1376 and HTB3 cells detected by 51Cr release assay. (h) Numbers of NK cells and transfected cells assessed by perforin polarization assay. (i, j) Conjugation assay showing the conjugates formed between NK cells and transfected HT-1376 (i) and HTB9 (j) cells. Experiments were conducted in triplicate. ^∗^*P* < 0.05, ^∗∗^*P* < 0.01, and ^∗∗∗^*P* < 0.001.

Our findings have elucidated the oncogenic role of circRHOT1 in BC progression and a novel circRHOT1/miR-3666/SMAD5 axis. To determinate whether circRHOT1 plays a role through this axis, we successfully introduced si-NC, si-circRHOT1#1, and si-circRHOT1#1+LV-SMAD5 into HT-1376 and HTB9 cells (Figures 7(a) and 7(b)). We found that the overexpression of SMAD5 could markedly rescue the suppression by circRHOT1 knockdown of cell proliferation (Figures 7(c)–7(e)) and migration (Figure 7(f)) according to the results from the CCK-8 assay (Figures 7(c) and 7(d)), EDU assay (Figure 7(e)), and Transwell migration assays (Figure 7(f)). We also observed that the SMAD5 overexpression could significantly reverse the aggravation by circRHOT1 knockdown of BC sensitivity to NK cells, as indicated by the results from the calcein release assay (Figure 7(g)), perforin polarization assay (Figure 7(h)), and conjugation assay (Figures 7(i) and 7(j)). The above results suggested that SMAD5 is crucial for the functional role of circRHOT1 in BC.

## 4. Discussion

In this study, we aimed to demonstrate whether circRHOT1 plays roles in BC progression. Our results showed that circRHOT1 was highly expressed in BC cells. The results from CCK-8, EDU, and Transwell migration assays suggested that the downregulation of circRHOT1 inhibited the proliferation and migration of BC cells. Furthermore, we observed that the NK cell-mediated cytotoxicity to circRHOT1 could dysregulate BC cells. The results also indicated that circRHOT1 knockdown could increase the sensitivity of BC cells to NK cells.

NK cells are the firstly verified subtypes of innate lymphoid cells (ILCs), which originated from lymphoid progenitor cells [26]. Numerous crucial functions of NK cells have recently been demonstrated, including exhibiting cell-killing function and an ability to produce proinflammatory cytokines in response to viral infection or to cell transformation [27–29]. The antitumor role and promising future of immunological therapy development have attracted wild interests from the scientific community. Accumulating evidence has revealed the essential role of NK cells in the development of a variety of cancers, such as hepatocellular carcinoma, breast cancer, prostate cancer, and renal carcinoma [30–33]. The function of NK cells in BC has also been investigated in-depth [25, 34, 35]. However, whether a circRNA can influence the NK cell-mediated cytotoxicity to bladder cancer remains to be uncovered. Here, our results showed the functional profile of circRHTO1 in tumorigenesis. It has been reported that there is a link between NK cell-mediated cellular progress and BC metastasis, especially in muscle cells [25, 36]. Here, our results suggested that circRHOT1 influenced both the migration and susceptibility of BC cells to NK cells. However, the mechanism underlying BC cell invasion has not been reported, and it is an ongoing investigation in our laboratory.

The upstream and downstream mechanisms of circRHOT1 in BC were investigated by bioinformatics analysis, followed by RNA pull-down, ChIP, and luciferase reporter gene assays. The results demonstrated that circRHOT1 expression in BC cells could be regulated by the transcription factor ZNF652. Transcriptional factors play a dispensable role in the circRNA expression profile [37, 38]. Our results indicated that the circRHOT1 expression could be induced by ZNF652 in BC cells, and this finding has enriched our knowledge on circRHOT1 expression in tumor cells. Subsequently, circRHOT1 was identified as a molecular sponge for miR-3666. Although the biological role of miR-3666 in multiple types of tumorigenesis has been widely reported [39–42], the role in BC progression or NK cells has not been investigated. Our results showed that miR-3666 overexpression could suppress BC cell proliferation, migration, and susceptibility to NK cells. Furthermore, our results showed that miR-3666 could target SMAD5 and negatively regulates SMAD5 expression in BC cells, suggesting that SMAD5 is essential for circRHOT1 to exhibit its functional role in BC progression. To our knowledge, the relationship between SMAD5 and NK cells remains unclear. Based on our results, SMAD5 may be a promising target for further exploring the biological functions of NK cells in BC progression.

Our findings suggest a novel ZNF652/cirRHOT1/miR-3666/SMAD5 axis in BC development; nevertheless, further studies are required in order confirm this finding. For instance, more tissue samples are needed in order to elucidate the clinical significance of circRHOT1 in BC. Extensive exploration of SMAD5 in BC progression and NK cell response is also required. Furthermore, the molecular mechanisms underlying the role of SMAD5 in BC progression remain to be uncovered. Overall, our study has partially demonstrated the biological and mechanical function of circRHTO1 in BC progression, which can be used as a new direction for further developing novel diagnostic or therapeutic targets for BC.

## Data Availability

The data used to support the findings of this study are included within the article.
